# Defect-Engineered La-Mn Co-Doped β-PbO_2_ Anodes for Energy-Efficient Zinc Electrowinning

**DOI:** 10.3390/ma19071370

**Published:** 2026-03-30

**Authors:** Yi Luo, Nan Li, Lingjing Yang, Jinlong Wei, Yuantao Yang, Wentao Wang, Yang Zhao, Ruidong Xu, Xuanbing Wang

**Affiliations:** 1 State Key Laboratory of Complex Nonferrous Metal Resources Clean Utilization, Kunming University of Science and Technology, Kunming 650093, China; 20232228007@stu.kust.edu.cn (Y.L.); linan19850226@126.com (N.L.); eslinjingyang@kust.edu.cn (L.Y.); weijinlong@stu.kust.edu.cn (J.W.); 20222102065@stu.kust.edu.cn (Y.Y.);; 2 Faculty of Metallurgical and Energy Engineering, Kunming University of Science and Technology, Kunming 650093, China

**Keywords:** zinc electrowinning, PbO_2_ anode, LaMn co-doping, interfacial kinetics

## Abstract

The high energy consumption of lead anodes in zinc production is caused by the slow oxygen evolution reaction (OER). We made a La-Mn co-doped β-PbO_2_ anode using electrodeposition to solve this issue. The XRD and XPS results show that adding La shrinks the lattice and changes the electron structure. This helps Mn^4+^ change into active Mn^3+^ and creates more active oxygen on the surface, making the reaction easier. EIS tests show that the charge transfer resistance (*R*_ct_) decreased by 4.2 times, decreasing from 147.6 Ω to 34.72 Ω at 1.0 V. The Bode phase peak also moved to a lower frequency (from 122 Hz to 0.215 Hz), proving that the electrochemically active surface area (ECSA) increased significantly. At the industrial current of 50 mA cm^−2^, the anode shows a low overpotential of 840 mV and a Tafel slope of 265 mV dec^−1^. This improved performance saves 187.10 kWh of energy per ton of zinc. Therefore, the LaMn-β-PbO_2_ anode is a promising and energy-saving option for industrial zinc production.

## 1. Introduction

Zinc is a vital non-ferrous metal widely utilized in galvanizing, alloys, and battery industries [1,2,3]. Currently, more than 80% of global zinc is produced via the hydromet-allurgical electrowinning process [4,5]. However, this process is notoriously energy-intensive, with the electricity cost accounting for approximately 40% of the total production expenditure [6]. The primary source of this high energy consumption is the significant overpotential of the oxygen evolution reaction (OER) occurring at the anode [7]. In industrial practice, traditional lead-silver (Pb-Ag) alloys are the most commonly used anodes due to their low cost [8,9]. Nevertheless, Pb-Ag anodes exhibit high OER overpotential and are prone to lead contamination in the cathode zinc, which severely limits the energy efficiency and product quality of the electrowinning process [10,11]. Consequently, developing novel anode materials with high catalytic activity and low overpotential, such as modified β-PbO_2_ electrodes, has become a critical research focus to achieve energy savings and cost reduction in the zinc hydrometallurgy industry [12].

Among various alternative materials, β-PbO_2_ has garnered extensive attention due to its high oxygen evolution overpotential, excellent electronic conductivity, and robust chemical stability in acidic electrolytes [13]. However, the electrocatalytic activity of pristine β-PbO_2_ remains insufficient for high-efficiency zinc electrowinning, primarily because of its limited active surface area and slow charge-transfer kinetics [11,14]. To address these issues, many researchers have employed doping strategies using metal ions or inert particles to modulate the physicochemical properties of β-PbO_2_. It has been reported that the introduction of foreign elements can refine the grain size, increase the electrochemically active surface area (ECSA), and optimize the electronic structure of the oxide matrix [15]. Transition metals like Manganese (Mn) are frequently chosen for their ability to provide active redox couples (e.g., Mn^3+^/Mn^4+^), which can act as catalytic centers to accelerate the OER process [16,17]. Nevertheless, single-element doping often reaches a plateau in performance improvement, necessitating more sophisticated strategies like co-doping to achieve superior catalytic efficiency [18].

The synergistic integration of rare earth (RE) elements and transition metals has become a prominent strategy for optimizing the performance of electrocatalysts [19]. Lanthanum (La), a typical RE element, possesses high oxophilicity and a significantly larger ionic radius (1.03 Å) compared to the host Pb^4+^ (0.775 Å) [20]. The incorporation of La into the rigid tetragonal β-PbO_2_ lattice induces localized lattice distortion and internal micro-stress due to this ionic size mismatch [21]. This structural perturbation breaks the symmetry of the Pb-O octahedra, facilitating the formation of structural defects and modulating the electronic density of states (DOS) [22]. These alterations increase the density of coordinatively unsaturated surface sites, which are essential for the adsorption and activation of water molecules [7].

The synergistic mechanism between La and Mn within the β-PbO_2_ matrix relies on the structural–electronic coupling effect [18]. We propose that the lattice strain induced by the larger La^3+^ ions modulates the local coordination environment and electronic configuration of the Mn centers. This structural perturbation facilitates the valence transition from Mn^4+^ to Mn^3+^. Consequently, the incorporation of lower-valence La^3+^ and Mn^3+^ species into the Pb^4+^ lattice necessitates a strategic charge compensation mechanism to maintain macroscopic electro-neutrality [23]. This process drives the profound enrichment of active surface oxygen species and coordinatively unsaturated sites, rather than mere structural vacancies. These anionic defects serve as key active sites, effectively optimizing the surface adsorption thermodynamics of OER intermediates on the β-PbO_2_ surface [7,11,22].

Kinetically, these surface oxygen vacancies function as pivotal active sites for the adsorption of oxygen-containing intermediates (OH*, O*, and OOH*) [23]. By modulating the local electronic structure, these defects optimize the binding affinity of the intermediates, thereby reducing the reaction energy barrier [24,25,26]. Furthermore, the presence of oxygen vacancies introduces donor levels within the bandgap, which enhances the intrinsic electrical conductivity of the β-PbO_2_ layer and accelerates electron transport from the electrolyte interface to the Pb-0.6% Ag alloy plates [27,28]. Additionally, these vacancy sites facilitate the deprotonation of water molecules, a step often identified as the rate-determining step (RDS) in acidic OER [29,30,31]. Consequently, the synergy between La-induced lattice distortion and Mn-based active centers, mediated by the abundant oxygen vacancies, establishes a kinetically favorable pathway for the four-electron OER process [32,33]. This structural and electronic modulation provides a solid rationale for the significant 4.2-fold reduction in charge transfer resistance *R*_ct_ confirmed by EIS analysis.

Herein, a La-Mn co-doped β-PbO_2_ anode was fabricated via a facile electrodeposition strategy to address the kinetic limitations of the OER in zinc electrowinning. Through systematic structural (XRD) and surface chemical (XPS) analyses, we elucidate a synergistic mechanism where La-induced lattice modulation promotes the Mn^4+^ to Mn^3+^ transition and the subsequent enrichment of surface oxygen vacancies V_O_. Electrochemical evaluations reveal that this defect engineering strategy yields a 4.2-fold reduction in charge transfer resistance *R*_ct_ from 147.6 Ω to 34.72 Ω and a 360 mV decrease in overpotential at 50 mA cm^−2^. More importantly, steady-state full-cell validations demonstrate a stable cell voltage reduction of 0.21 V, which translates to a precise energy saving of 187.10 kWh per ton of zinc, validating the industrial viability of the proposed anode for energy-efficient hydrometallurgy.

## 2. Materials

### 2.1. Experimental Principles

Lead oxide (PbO), lead nitrate (Pb(NO_3_)_2_), sodium hydroxide (NaOH, flakes), sodium citrate (C_6_H_5_Na_3_O_7_), lanthanum nitrate hexahydrate (La(NO_3_)_3_·6H_2_O), manganese nitrate hydrate (Mn(NO_3_)_2_·4H_2_O), and zinc sulfate heptahydrate (ZnSO_4_·7H_2_O) were purchased from Shanghai Aladdin Biochemical Technology Co., Ltd., Shanghai, China. Nitric acid (HNO_3_) and sulfuric acid (H_2_SO_4_) were supplied by Chongqing Chuandong Chemical (Group) Co., Ltd., Chongqing, China. All chemical reagents used in this work were of analytical grade and used without any further purification. All aqueous solutions were prepared with deionized water.

### 2.2. Preparation Processes of Electrodes

Pretreatment of substrate and electrodeposition of α-PbO_2_ interlayer: Commercial Pb-0.6% Ag alloy plates with dimensions of 1 cm × 2 cm were employed as the substrate. Prior to electrodeposition, the substrates were mechanically polished to remove the surface passivated layer and expose a fresh metallic surface, followed by thorough rinsing. The α-PbO_2_ intermediate layer was fabricated via anodic electrodeposition in a standard two-electrode cell, where the pretreated Pb-Ag plate served as the anode and a stainless steel plate acted as the cathode. The alkaline electrolyte consisted of 33.5 g L^−1^ PbO dissolved in 120 g L^−1^ NaOH solution. A specific two-step galvanostatic deposition strategy was employed: an initial high current density of 200 mA cm^−2^ was applied for 30 s to rapidly oxidize the substrate until the surface color turned from silver-grey to black. Immediately thereafter, the current density was reduced to 15 mA cm^−2^ for continuous oxidation and growth over 50 min. This process was maintained at 40 °C with a constant magnetic stirring speed of 150 rpm.

Electrodeposition of La-Mn co-doped β-PbO_2_ active layer: Subsequently, the La-Mn co-doped β-PbO_2_ active layer was anodically deposited onto the obtained Pb-Ag/α-PbO_2_ electrode using the same two-electrode system. The acidic electroplating bath contained 250 g L^−1^ Pb(NO_3_)_2_, 50 g L^−1^ Mn(NO_3_)_2_, 7 g L-1 La(NO_3_)_3_, and 1 g L^−1^ sodium citrate as an additive. The pH of the solution was strictly adjusted to 1.0 using concentrated HNO_3_. The electrodeposition was conducted at a current density of 10 mA cm^−2^ for 60 min. The bath temperature was kept at 40 °C under continuous magnetic stirring at 300 rpm. After deposition, the prepared composite anode (denoted as LaMn-PbO_2_) was thoroughly rinsed with deionized water and dried in air.

Preparation of control samples: For comparison, pristine β-PbO_2_ and single Mn-doped β-PbO_2_ (denoted as Mn-PbO_2_) electrodes were fabricated under identical electrodeposition conditions. Specifically, the pristine β-PbO_2_ electrode was deposited from the acidic plating bath without the addition of both La(NO_3_)_3_ and Mn(NO_3_)_2_. Similarly, the Mn-PbO_2_ control sample was prepared by omitting only La(NO_3_)_3_ from the standard co-deposition bath.

### 2.3. Physical Characterization

The crystal structures of the prepared electrodes were characterized by an X-ray diffractometer (XRD, D/MAX-2200, Rigaku, Tokyo, Japan) equipped with monochromatic Cu-Ka radiation (λ = 0.154 nm). The XRD patterns were collected in the 2θ range of 20° to 80° with a scanning rate of 5° min^−1^. Surface morphologies and elemental distributions were observed using a field emission scanning electron microscope (NanoSEM-450, FEI, Hillsboro, OR, USA) equipped with an energy dispersive spectrometer (EDS). Furthermore, the surface chemical states were investigated using an X-ray photoelectron spectrometer (XPS, PHI 5500, Physical Electronics, Chanhassen, MN, USA) equipped with an Al Kα X-ray source.

### 2.4. Electrochemical Tests

A series of experiments were conducted in the electrolyte containing 50 g L^–1^ Zn^2+^ and 150 g L^–1^ H_2_SO_4_ to investigate the OER catalytic performance using a three-electrode setup. All the electrochemical tests were performed at 35 °C. The as-prepared composite anode was used as a working electrode. A stainless-steel plate served as the counter electrode and a saturated mercurous sulfate electrode (MSE) served as the reference electrode. The linear sweep voltammetry (LSV) and cyclic voltammetry (CV) were recorded with a scanning rate of 5 mV s^–1^. The testing frequency of AC impedance ranged from 100,000 to 0.1 Hz. Notably, all polarization curves and overpotential values are presented without iR-compensation to realistically evaluate the apparent performance and actual energy consumption of the anodes under industrial-scale zinc electrowinning conditions.

## 3. Results and Discussion

### 3.1. Structural Evolution of La-Mn Co-Doped β-PbO_2_

**Figure 1:**
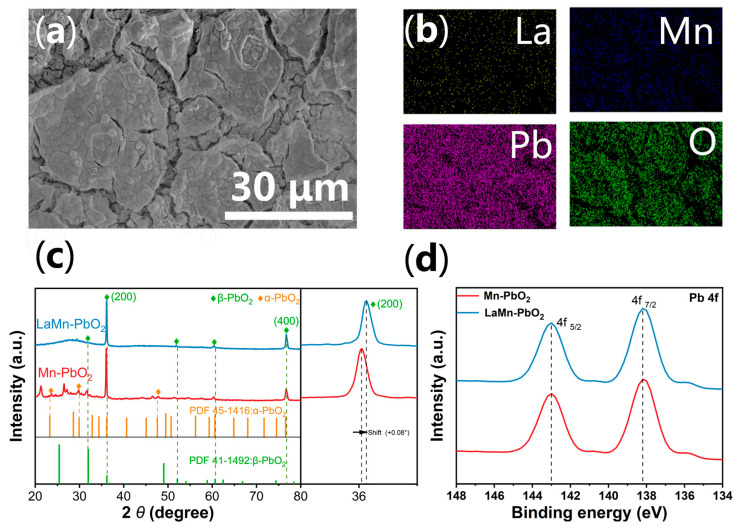
(**a**) SEM image of the LaMn-PbO_2_ electrode; (**b**) EDS elemental mapping images of La, Mn, Pb, and O; (**c**) XRD patterns of the Mn-PbO_2_, and LaMn-PbO_2_ electrodes; (**d**) XPS Pb 4f spectra of the Mn-PbO_2_ and LaMn-PbO_2_ electrodes. The vertical dashed lines in (**c**,**d**) are provided to highlight the shifts of the corresponding XRD diffraction peaks and XPS binding energies, respectively.

Figure 1a reveals the surface morphology of the La-Mn co-doped β-PbO_2_ electrode, which exhibits a typical “mud-crack” structure. The surface consists of large, irregular domains separated by deep cracks, a characteristic feature attributed to the release of internal lattice stress accumulated during the anodic electrodeposition process. Figure 1b confirms the elemental homogeneity of the coating through EDS mapping. More critically, quantitative analysis highlights a substantial variation in chemical composition: the introduction of Lanthanum nearly doubles the manganese incorporation, with the Mn atomic concentration rising from 8.3% in the Mn-PbO_2_ electrode to 16.7% in the LaMn-PbO_2_ electrode (see Supporting Information). Figure 1c presents the XRD patterns of the prepared electrodes. The Mn-PbO_2_ and LaMn-PbO_2_ electrodes exhibit characteristic diffraction peaks at 32.0°, 36.2°, 53°, 61° and 76.2°, which correspond to the (101), (200), (220), (112), and (400) planes of tetragonal rutile β-PbO_2_ (JCPDS No. 41-1492), respectively. However, the LaMn-PbO_2_ spectrum shows a marked crystallographic evolution: the peak associated with the (101) plane is suppressed, while the (200) and (400) reflections become dominant, indicating a strong preferred orientation along the a-axis induced by La doping. Detailed quantitative analysis of the primary (200) plane (Figure 1c, right panel) reveals an anomalous lattice contraction, evidenced by a positive peak shift of 0.08° relative to the Mn-PbO_2_ electrode. Correspondingly, the lattice parameter a contracts by 0.011 Å. This macroscopic contraction is the synergistic physical consequence of the stoichiometric disparity and the defect-induced framework collapse. Based on the Kröger-Vink defect chemistry, the substitution of Pb^4+^ by La^3+^ ($La_{Pb}^{\prime }$) triggers the spontaneous generation of oxygen vacancies ($V_{O}^{\cdot \cdot }$) to maintain electroneutrality (2 $La_{Pb}^{\prime }$ + $V_{O}^{\cdot \cdot }$ = 0). As quantified by EDS, the surge in manganese content (from 8.3% to 16.7%) confirms that the incorporation of smaller Mn ions (Mn^4+^: ~0.53 Å, Mn^3+^: ~0.58 Å) vastly outnumbers the larger La^3+^: (~1.03 Å) dopants. This high-frequency substitution statistically overrides the localized steric expansion from La. Furthermore, the escape of lattice oxygen atoms to form vacancies creates voids that lead to a localized structural relaxation and collapse, which collaboratively drives the overall 0.011 Å contraction of the α-axis. This structural contraction is directly driven by the aforementioned surge in manganese content (from 8.3% to 16.7%): the extensive substitution of smaller radii Mn ions Mn^4+^/Mn^3+^ into the Pb^4+^ sites overrides the steric expansion typically expected from the larger La^3+^ dopants. This proves that the structural distortion mentioned earlier manifests macroscopically as a lattice contraction, which tightens the atomic distances and facilitates faster electron transfer. Additionally, the FWHM broadening of the (200) peak confirms that this intense co-doping effectively refines the grain size. Furthermore, based on the Scherrer equation, the FWHM broadening of the (200) peak indicates a reduction in the average crystallite size from 28.5 nm to 27.3 nm. This confirms that the intense co-doping effectively refines the grains at the crystallographic level.

Finally, the chemical stability of the host lattice was probed using XPS. Figure 1d displays the high-resolution Pb 4f spectra, featuring symmetric doublets at 137.6 eV (Pb 4f_7/2_) and 142.5 eV (Pb 4f_5/2_). Despite the significant lattice distortion and the drastic increase in Mn concentration, the binding energies of the Pb 4f peaks remain virtually unchanged. This confirms that the coordination environment of the bulk Pb atoms is preserved, demonstrating that the β-PbO_2_ matrix maintains robust structural integrity even under high-concentration doping conditions [34].

**Figure 2:**
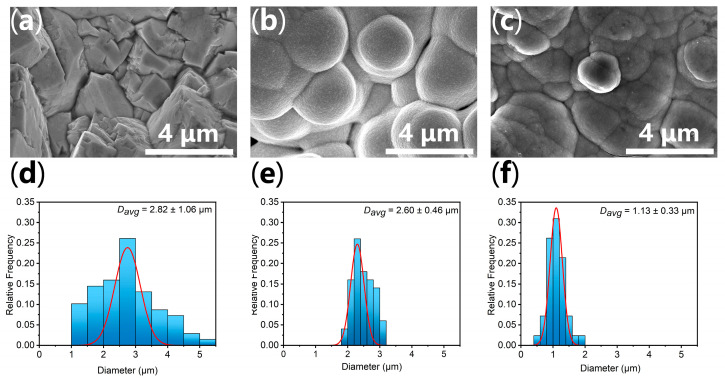
(**a**) SEM images of the β-PbO_2_, (**b**) Mn-PbO_2_, and (**c**) LaMn-PbO_2_ electrodes; corresponding grain size distribution histograms of the (**d**) β-PbO_2_, (**e**) Mn-PbO_2_, and (**f**) LaMn-PbO_2_ electrodes. The blue boxes represent the statistical histogram of the size distribution, and the red line is the corresponding fitted Gaussian distribution curve.

To further investigate the influence of doping on the microscopic morphology, high-magnification scanning electron microscopy (SEM) and corresponding grain size statistical analyses were conducted (Figure 2). The β-PbO_2_ electrode (Figure 2a) exhibits a typical coarse surface composed of large, pyramid-shaped crystals with sharp edges. Upon Mn doping (Figure 2b), the crystal growth habit undergoes a distinct transition, forming relatively regular and closely packed spherical grains. With the further co-doping of La (Figure 2c), these regular spheres undergo a structural evolution, presenting a collapsed spherical morphology that results in a noticeably rougher surface texture.

The corresponding grain size distribution histograms (Figure 2d–f) quantitatively confirm this morphological refinement. The average macroscopic grain size (*D*_avg_) progressively decreases from 2.82 ± 1.06 μm for the β-PbO_2_ electrode to 2.60 ± 0.46 μm for the Mn-PbO_2_ electrode. Strikingly, the LaMn-PbO_2_ electrode exhibits a drastic reduction in *D*_avg_ to 1.13 ± 0.33 μm, accompanied by a significantly narrower size distribution. This substantial refinement indicates that the LaMn co-doping synergistically inhibits excessive crystal growth and promotes nucleation during electrodeposition. This macroscopic observation perfectly corroborates the crystallite size reduction previously deduced from the XRD Scherrer analysis. Such a refined, highly uniform, and collapsed micro-spherical architecture is highly advantageous, as it significantly increases the physical contact area between the electrode and the electrolyte, which is highly conducive to exposing abundant accessible active sites for the oxygen evolution reaction.

### 3.2. Surface Chemical States and Electronic Structure

**Figure 3:**
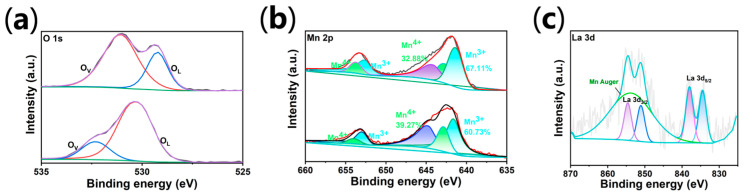
High-resolution XPS spectra of the prepared anodes: (**a**) O 1s for the LaMn-PbO_2_ electrode and Mn-PbO_2_ electrode, (**b**) Mn 2p for the LaMn-PbO_2_ electrode and Mn-PbO_2_ electrode, and (**c**) La 3d for the LaMn-PbO_2_ electrode. In the spectra, the differently colored lines and shaded areas represent the raw experimental data, the overall fitted curves, the backgrounds, and the deconvoluted peaks corresponding to various chemical states (as labeled), respectively.

High-resolution X-ray photoelectron spectroscopy (XPS) was employed to systematically decouple the surface electronic structure and defect chemistry of the prepared anodes (Figure 3). The survey spectra confirm the presence of Pb, Mn, and O in both samples, while the characteristic La reflections in the co-doped spectrum validate the successful lattice integration of the rare-earth species. As illustrated in Figure 3c, the La 3d spectrum of the LaMn-PbO_2_ electrode exhibits two primary spin–orbit doublets at 834.9 eV (3d_5/2_) and 851.7 eV (3d_3/2_), separated by a splitting energy of 16.8 eV, confirming the La^3+^ oxidation state [18,20,35]. The prominent satellite peaks originate from intense charge-transfer interactions from the ligand O 2p orbitals to the empty La 4f orbitals, indicating a strong electronic coupling between the La dopants and the oxide matrix.

The role of manganese valence states is a decisive factor in catalytic activity. A vertical comparison between the Mn 2p spectra of the two electrodes (Figure 3b) reveals a profound valence reconstruction. The rich chemistry of manganese oxides heavily relies on the reversible redox couple of Mn^4+^/Mn^3+^, which is widely recognized as a primary driver for enhanced electrochemical kinetics and surface reactivity [36]. For the single Mn-doped electrode, the surface is predominantly occupied by Mn^4+^ species (~39%). However, the introduction of La triggers a significant valence inversion in the LaMn-PbO_2_ sample, where the Mn^3+^ proportion increases markedly from 60.73% to 67.11%. From a mechanistic standpoint, this enrichment of Mn^3+^ (e g^−1^ configuration) is highly favorable, as it optimizes the orbital symmetry for the adsorption of oxygenated intermediates [37].

Crucially, the fundamental origin of this Mn^4+^ to Mn^3+^ reduction is intrinsically linked to the defect chemistry provoked by La doping, which is perfectly substantiated by the O 1s spectra (Figure 3a). This aligns well with recent findings that tailored electrodeposition and structural cross-linking in manganese oxides can fundamentally alter their local defect configurations, thereby significantly boosting their interfacial charge transfer and electrocatalytic potential [38]. The underlying defect chemistry of this valence shift can be strictly elucidated by Kröger-Vink notation. When the lower-valence La^3+^ ion substitutes the host Pb^4+^ site, it induces negative effective charges ($La_{Pb}^{\prime }$), which are compensated by the spontaneous generation of oxygen vacancies ($V_{O}^{\cdot \cdot }$) to maintain macroscopic electroneutrality: 2 $La_{Pb}^{\prime }$ + $V_{O}^{\cdot \cdot }$ = 0. Each formed vacancy subsequently releases two localized electrons into the lattice ($V_{O}^{X}\to V_{O}^{\cdot \cdot }+2e^{\prime }$). These electrons are preferentially captured by the adjacent electroactive Mn species (2$e^{\prime }$ + 2Mn^4+^ → 2Mn^3+^), facilitating the observed valence inversion. The deconvoluted O 1s profile of the LaMn-PbO_2_ electrode demonstrates a drastic elevation in the active surface oxygen species (O_ads_, ~531.5 eV) peak intensity compared to the single-doped counterpart [35]. This specific signal represents oxygen atoms residing adjacent to the newly formed V_O_ sites; the loss of a neighboring oxygen atom significantly reduces the local electron cloud density and the electronic shielding effect, thereby shifting the binding energy to a higher value. When the lower-valence and larger-radius La^3+^ ion substitutes the host Pb^4+^ site, it inevitably induces severe lattice distortion and a local positive charge deficit. To maintain macroscopic electroneutrality and release the internal strain, the oxide lattice spontaneously reconstructs, generating a abundant amount of coordinatively unsaturated sites and adsorbed active oxygen species. During this surface reconstruction, the localized electrons left behind in the lattice are preferentially captured by the adjacent electroactive Mn species, thereby driving the widespread reduction of Mn^4+^ to Mn^3+^. Consequently, the La-induced generation of abundant active surface oxygen species fulfills a pivotal dual role: first, they act as highly active chemisorption centers to trap water molecules and form a hydrophilic *OH adlayer (accelerating the initial dissociation step); second, they modulate the local electronic environment to stabilize the highly active Mn^3+^ species. This defect-driven synergistic mechanism provides a robust electronic foundation for the superior OER kinetics observed in subsequent electrochemical evaluations.

### 3.3. Electrochemical Performance of Composite Electrodes

**Figure 4:**
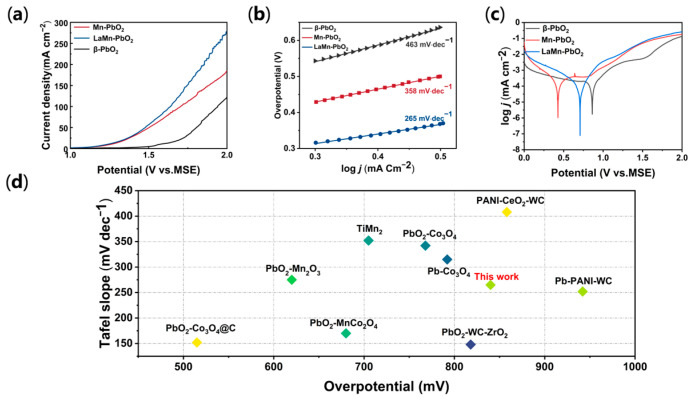
Electrocatalytic OER performance of the pristine β-PbO_2_, Mn-PbO_2_, and LaMn-PbO_2_ electrodes in 50 g L^–1^ Zn^2+^ and 150 g L^–1^ H_2_SO_4_: (**a**) linear sweep voltammetry (LSV) polarization curves at 2 mV s^−1^; (**b**) the corresponding Tafel plots; (**c**) potentiodynamic polarization curves at 2 mV s^−1^; (**d**) performance comparison (Tafel slope vs. overpotential) of the LaMn-PbO_2_ anode with other recently reported Pb-based OER electrocatalysts.

To evaluate the electrocatalytic activity towards the oxygen evolution reaction (OER), linear sweep voltammetry (LSV) measurements were conducted in a simulated zinc electrowinning electrolyte (50 g L^–1^ Zn^2+^ and 150 g L^–1^ H_2_SO_4_). As depicted in Figure 4a, the anodic current densities for all samples exhibit a monotonic increase driven by the applied potential. However, the pristine β-PbO_2_ electrode displays sluggish OER kinetics, requiring a substantial anodic potential to initiate the reaction. Upon single Mn doping, the polarization curve exhibits a distinct cathodic shift, indicating a preliminary enhancement in catalytic activity. Most notably, the La-Mn co-doped LaMn-PbO_2_ electrode delivers the highest current density across the entire potential range. At the benchmark current density of 50 mA cm^−2^ (a typical operating condition for metal electrowinning), the LaMn-PbO_2_ anode achieves a remarkably low overpotential of 840 mV. This substantial reduction in overpotential can be ascribed to the synergistic interplay of two factors: the expanded electrochemically active surface area (ECSA) induced by the modified micro-architecture, and the optimized electronic configuration enriched with catalytic Mn^3+^ sites, which effectively lowers the kinetic activation barrier for water oxidation. To quantitatively decipher the reaction kinetics, Tafel plots were constructed (Figure 4b). The Tafel slopes for the pristine β-PbO_2_, Mn-PbO_2_, and LaMn-PbO_2_ electrodes are calculated as 463 mV dec^−1^, 358 mV dec^−1^, and 265 mV dec^−1^, respectively. The LaMn-PbO_2_ electrode exhibits the minimum Tafel slope, unambiguously confirming its superior intrinsic OER kinetics in the highly acidic medium. When the Tafel slope exceeded 120 mV dec^–1^, the OER rate-limited step was determined by step—the formation and adsorption of the first intermediate. The OER by decomposition of water in acidic media can be described with the following formula:

2H_2_O − 4e^−^→4H^+^ + O_2_ (1)

The OER steps can be elaborated as

S + H_2_O → S − OH_ads_ + H^+^ + e^−^ (2)

S − OH_ads_ → S − O_ads_ + H^+^ + e^−^ (3)

2S − OH_ads_ → S − O_ads_ + S + H_2_O (4)

S − O_ads_ → S + 1/2 O_2_ (5)

The experimentally determined Tafel slope of the La-Mn co-doped electrode (265 mV dec^–1^) is significantly lower than that of the reference samples, although it remains in a range indicative of the first electron transfer step being rate-determining. This quantitative reduction in the Tafel slope suggests that the LaMn co-doping strategy effectively modulates the adsorption energy of the intermediates on the electrode surface, thereby facilitating the initial water dissociation step and accelerating the overall reaction rate.

Finally, to position the electrochemical performance of the prepared material within the current state of the art, the LaMn-PbO_2_ anode was benchmarked against other representative Pb-based anodes recently reported in the literature. As visualized in the scatter plot of Figure 4d, the “This work” data point is located in the favorable lower-left region, which corresponds to a simultaneous achievement of low overpotential and optimized reaction kinetics. This comparison highlights that the LaMn-PbO_2_ electrode exhibits a competitive balance between energy efficiency and catalytic rate, which is kinetically superior or comparable to several advanced composite anodes such as PbO_2_-Co_3_O_4_, TiMn_2_, and Pb-PANI-WC [6,39,40,41,42,43]. Consequently, these results convincingly demonstrate that the La-Mn co-doped β-PbO_2_ composite electrode is a highly promising candidate for energy-saving applications in the zinc electrowinning industry.

### 3.4. Interfacial Kinetics and Charge Transfer

**Figure 5:**
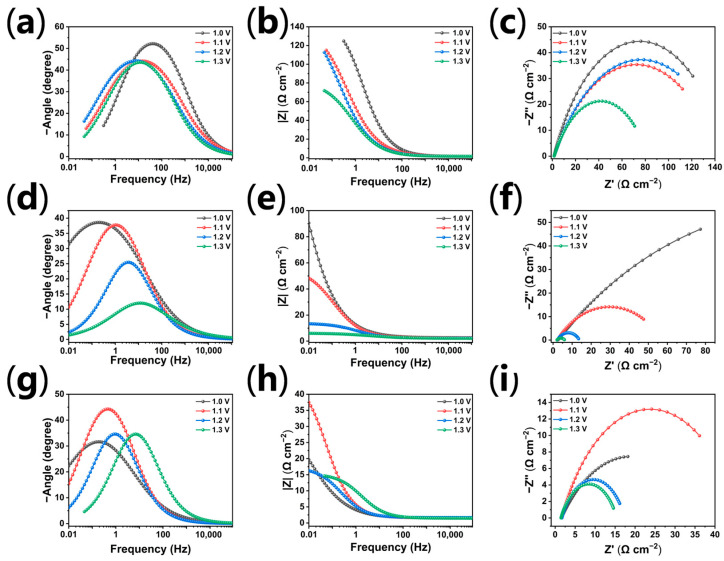
EIS spectra of the prepared electrodes measured at various applied potentials in 50 g L^–1^ Zn^2+^ and 150 g L^–1^ H_2_SO_4_: (**a**–**c**) β-PbO_2_, (**d**–**f**) Mn-PbO_2_, and (**g**–**i**) LaMn-PbO_2_ electrodes at various potentials; (**a**,**d**,**g**) Nyquist plots, (**b**,**e**,**h**) Bode |Z| plots, and (**c**,**f**,**i**) Bode phase plots.

To quantitatively evaluate the interfacial oxygen evolution reaction (OER) kinetics, steady-state electrochemical impedance spectroscopy (EIS) measurements were systematically conducted across a highly anodic potential window from 1.0 V to 1.3 V (Figure 5). The resulting Nyquist plots (Figure 5a,d,g) were meticulously fitted using an *R*_s_(Q*R*_ct_) equivalent. As the applied potential increases, the apparent charge-transfer resistance (*R*_ct_) values for all electrodes exhibit a distinct downward trend, reflecting the exponential acceleration of charge transfer driven by the elevated electrochemical overpotential. Specifically, at the kinetic onset potential of 1.0 V, the *R*_ct_ values for pristine β-PbO_2_, Mn-PbO_2_, and LaMn-PbO_2_ are mathematically extracted as 147.6 Ω, 264.2 Ω, and 34.72 Ω, respectively. It is noteworthy that in the kinetically controlled region (1.0 V), the onset kinetics of the single-doped Mn-PbO_2_ electrode were not improved as expected; instead, its apparent charge-transfer resistance (*R*_ct_) increased to 264.2 Ω. Combined with the XPS analysis, this can be primarily attributed to the singular incorporation of high-valence Mn^4+^ (approximately 39%). Without triggering sufficient oxygen vacancies for charge compensation, this aliovalent substitution likely disrupts the local conductive network of the rutile lattice. However, this kinetic sluggishness is fundamentally reversed by the synergistic LaMn co-doping strategy, which drastically reduces the *R*_ct_ of LaMn-PbO_2_ to 34.72 Ω. Furthermore, it is highly significant that the LaMn-PbO_2_ electrode simultaneously exhibits an optimally minimized high-frequency intercept (*R*_s_) of 1.52 Ω. This demonstrates that the synergistic La-Mn codoping not only accelerates interfacial charge transfer but also effectively enhances the intrinsic electrical conductivity of the active coating, which is crucial for minimizing parasitic ohmic energy loss in actual industrial operations without iR-compensation. To further decouple this significant performance leap, the relaxation time constants at the interface were extracted from the Bode phase plots (Figure 5c,f,i). At 1.0 V, the characteristic phase peak of LaMn-PbO_2_ markedly shifts to an ultra-low frequency (*f*_max_ = 0.215 Hz). Considering the highly defective surface and the redox-active nature of the La-Mn co-doped electrode, a constant phase element (CPE) was employed in the equivalent circuit instead of an ideal capacitor. The relationship is defined as follows:

*τ* = *R*_ct_ *C*_eff_ = 1/(2π *f*_max_) (6)

This pronounced frequency displacement mathematically dictates a considerable expansion in the effective capacitance (C_eff_). This *C*_eff_ represents the synergistic macroscopic contribution of both the electrostatic double-layer capacitance and the massive defect-induced faradaic pseudocapacitance. As detailed in the fitting data (Table S1), the *C*_eff_ of LaMn-PbO_2_ reaches 21.32 mF, which is over 2400 times higher than that of the pristine β-PbO_2_ (0.0088 mF). This robust capacitive evidence unambiguously confirms that the abundant mixed-valence manganese species (Mn^3+^/Mn^4+^) and highly concentrated surface oxygen vacancies act as highly active redox centers, triggering intense local Faradaic pseudocapacitive reactions. Together with the profoundly expanded electrochemically active surface area (ECSA) caused by the La-induced morphological collapse, they collaboratively eliminate the initial kinetic barrier for water oxidation.

More intriguingly, a pseudo-impedance inversion emerges in the high-overpotential regime (≥1.2 V), where the steady-state *R*_ct_ of LaMn-PbO_2_ slightly surpasses that of Mn-PbO_2_. This phenomenon stems not from a decay in intrinsic catalytic activity, but rather from a secondary mass-transport limitation triggered by the highly vigorous oxygen evolution kinetics. The intense OER process under high driving forces generates copious amounts of oxygen gas. The highly rough, ‘mud-crack’ micro-architecture with very obvious cracks on LaMn-PbO_2_ is highly susceptible to trapping these macroscopic bubbles, consequently forming a transient, insulating gas-shielding layer. This dynamic bubble coverage effect is perfectly corroborated by the high-potential *C*_eff_ data: as the applied potential increases to 1.3 V, the *C*_eff_ of LaMn-PbO_2_ experiences a precipitous drop from 21.32 mF to a mere 1.68 mF (significantly lower than the 6.91 mF of Mn-PbO_2_ at the same potential). Since the effective capacitance (*C*_eff_) is strongly dependent on the accessible solid–liquid contact area and the exposed redox-active sites, this drastic capacitive shrinkage serves as direct quantitative evidence that the bubble shielding layer physically obstructs these intrinsic active centers. Therefore, the impedance and capacitive responses at the low-potential stage serve as the most accurate descriptors for evaluating the intrinsic catalytic superiority of this composite anode.

Furthermore, as the anodic driving force intensifies from 1.0 V to 1.3 V, the phase peak (*f*_max_) of the LaMn-PbO_2_ electrode progressively shifts toward higher frequencies (reaching 6.81 Hz at 1.3 V, Figure 5i). This positive frequency displacement signifies a continuous reduction in the relaxation time (τ), unequivocally demonstrating that the elevated electric field significantly accelerates the intrinsic catalytic turnover and electron exchange rate of the adsorbed oxygenated intermediates (e.g., *OH and *O). In summary, the synergistic combination of abundant electroactive sites (including both the expanded ECSA and the highly reactive pseudocapacitive centers, as evidenced by the giant *C*_eff_) and accelerated interfacial electron exchange kinetics (evidenced by the potential-driven peak displacement) comprehensively establishes the outstanding OER catalytic mechanism of the LaMn-PbO_2_ electrode.

### 3.5. Energy Consumption Assessment and Industrial Application Potential

In industrial hydrometallurgy, full-cell zinc electrowinning involves complex cathodic deposition dynamics and long-term pilot-scale operations; while multi-month pilot-scale testing is beyond the scope of this fundamental electrocatalytic study, the macroscopic energy-saving potential and long-term viability of the prepared anodes can be rigorously projected based on steady-state full-cell validations and accelerated aging tests, rather than solely relying on transient half-cell overpotentials.

According to the fundamental principles of electrolysis, the specific DC energy consumption (W, kWh t^−1^) for producing one ton of zinc can be calculated using the following equation:

*W* = (*V*⋅*I*⋅*t* × 1000)/(*q*⋅*I*⋅*t*⋅η) = (*V* × 1000)/(*q*⋅η) = 819.67*V/η* (7)

where W is the electrical energy (kW·h); I is the current (A); t is the electrodeposition time (h); V is the cell voltage (V); q is the electrochemical equivalent of zinc (1.220 g (A^–1^ h^–1^)); η is the current efficiency (%).

**Figure 6:**
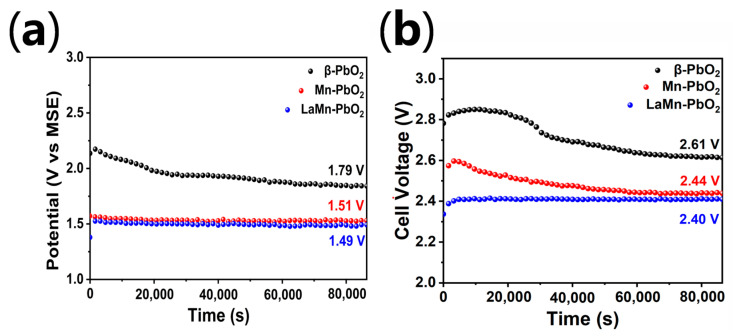
Long-term electrochemical stability of the prepared electrodes during a 24 h continuous operation at an industrial current density of 50 mA cm^−2^: (**a**) anodic chronopotentiometric curves in the half-cell configuration and (**b**) full-cell voltage profiles.

Under identical electrolysis conditions (same electrolyte composition, temperature, electrode spacing, and cathode material), the difference in cell voltage between different anode materials is primarily governed by the difference in their anodic oxygen evolution overpotential. While the initial LSV results (Figure 4a) provide fundamental kinetic insights, industrial electrowinning is strictly a long-term steady-state process. To authentically evaluate this, 24 h continuous chronopotentiometry (CP) and full-cell voltage tests were conducted at the industrial current density of 50 mA cm^−2^ (Figure 6).

The long-term steady-state tests perfectly corroborate the transient LSV evaluations. Remarkably, for the LaMn-co-doped anode, the steady-state anodic potential (~1.49 V) matches its initial LSV performance (1.49 V) almost seamlessly. This remarkable consistency confirms that the highly hydrophilic nature of the LaMn-doped surface enables instantaneous electrolyte penetration and immediate peak performance. In contrast, the pure β-PbO_2_ electrode captures a significantly higher potential in the fast LSV scan (1.80 V) than its final stabilized CP voltage (~1.57 V), visually capturing its sluggish in situ wetting and activation process. The remarkably steady voltage profile of the LaMn-PbO_2_ electrode demonstrates its excellent electrochemical stability and sustained catalytic activity under continuous industrial current density.

Based on the steady-state voltage difference confirmed by the full-cell tests, a realistic projection of the energy savings was conducted. Based on this confirmed voltage reduction of 0.21 V, and assuming a typical industrial current efficiency of 92% (η = 0.92$), the application of the La-Mn co-doped anode reduces the electricity consumption by approximately 187.10 kW·h per ton of zinc compared to the pure β-PbO_2_ anode.

**Figure 7:**
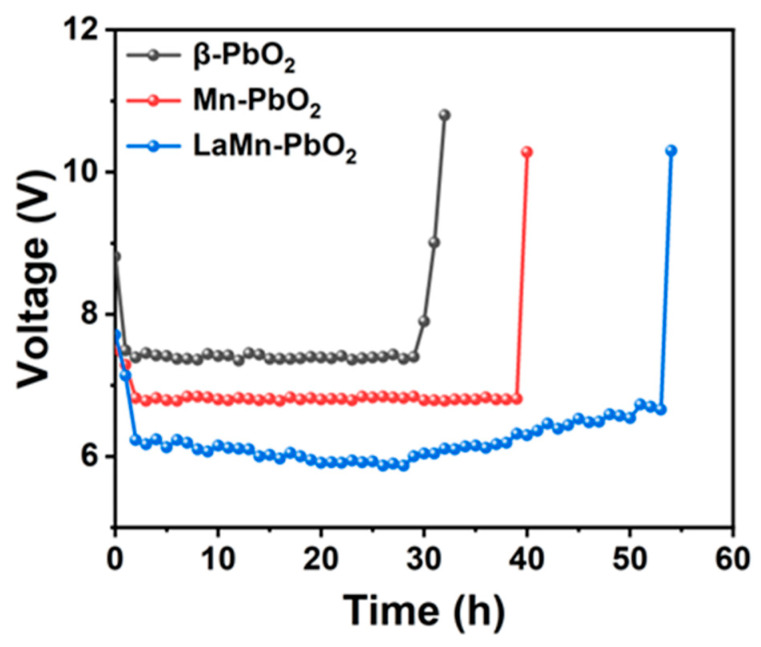
Accelerated service life tests of the prepared anodes conducted at a high current density of 1.5 A cm^−2^ to evaluate the long-term electrochemical durability and corrosion resistance.

Furthermore, accelerated service life tests were performed under extreme conditions (Figure 7) to evaluate structural durability.

Despite the severe physical stress and corrosive environment at high current density, the LaMn-co-doped anode maintained its structural integrity for 54 h, significantly exceeding the 32 h figure for the pure β-PbO_2_ anode. This demonstrates that the LaMn-doping effectively strengthens the coating-substrate adhesion and enhances the resistance to electrochemical corrosion. Consequently, the prepared anode offers a promising combination of stable energy savings and robust corrosion resistance for industrial zinc electrowinning.

In summary, this chapter systematically evaluates the industrial application potential of the La-Mn co-doped β-PbO_2_ anode through steady-state cell voltage measurements and accelerated service life tests. The results demonstrate that the co-doped anode exhibits superior electrochemical stability during continuous operation at 50 mA cm^−2^, achieving a significant cell voltage reduction of 0.21 V (2.61 V to 2.40 V) compared to the pure β-PbO_2_ anode. Based on Faraday’s law of electrolysis, this reduction translates into a substantial DC energy saving of 187.10 kWh per ton of zinc produced. Furthermore, the significantly extended duration in accelerated service life tests confirms the enhanced structural durability and corrosion resistance of the La-Mn co-doped coating. Combining high energy efficiency with robust operational stability, the prepared anode represents a highly promising and sustainable candidate for the next-generation green zinc electrowinning industry.

## 4. Conclusions

In summary, a novel La-Mn co-doped β-PbO_2_ composite electrode with an α-PbO_2_ intermediate layer was successfully fabricated via an electrodeposition method. This hierarchical architecture ensures excellent bonding strength and conductivity between the substrate and the functional coating. Physicochemical characterizations revealed that the introduction of La induced a strategic morphological transition to a collapsed architecture, significantly enlarging the electrochemically active surface area. Furthermore, the doping strategy effectively modulated the electronic structure, promoting the enrichment of Mn^3+^ active species and the generation of high-density active surface oxygen species, which served as key active centers to facilitate the oxygen evolution reaction. Most significantly, systematic EIS analysis successfully decoupled the interfacial dynamics, revealing a fundamental acceleration in OER kinetics evidenced by a 4.2-fold reduction in charge transfer resistance (*R*_ct_) at 1.0 V. The notable low-frequency shift of the Bode phase peak (from 122 Hz to 0.215 Hz) further substantiates a substantial expansion of the active interface and a significantly optimized relaxation time constant, facilitating an accelerated turnover frequency for water oxidation. Benefiting from these interfacial advantages, the LaMn-PbO_2_ electrode delivered a superior electrochemical performance with a lower overpotential of 840 mV at 50 mA cm^−2^ and a reduced Tafel slope of 265 mV dec^−1^. In the simulated zinc electrowinning process, this kinetic superiority translates into a substantial theoretical energy saving of 187.10 kWh per ton of zinc. Therefore, this work provides a promising anode candidate driven by optimized interfacial kinetics for industrial acidic OER processes.

## Data Availability

The original contributions presented in this study are included in the article/Supplementary Material. Further inquiries can be directed to the corresponding authors.

## Supplementary Materials

The following supporting information can be downloaded at https://www.mdpi.com/article/10.3390/ma19071370/s1. Figure S1. EDS elemental mapping (Pb, O, Mn, and La) of (a) β-PbO_2_, (b) Mn-PbO_2_, and (c) LaMn-PbO_2_ electrodes. Table S1. Comparison of interfacial capacitance parameters (*C*_eff_) for the prepared electrodes in acidic zinc electrowinning electrolyte. Table S2. Fitting parameters of the equivalent circuit (*R*_s_(Q*R*_ct_)) for different anodes at various anodic potentials in the zinc electrowinning electrolyte. Table S3. Characteristic frequency values (*f*_max_) determined from Bode phase plots for different anodes at various anodic potentials in the zinc electrowinning electrolyte. Table S4. corresponding relaxation time constants (τ) for the prepared anodes at different anodic potentials. in the zinc electrowinning electrolyte. Table S5. Proposed electrochemical deposition mechanism of PbO_2_ active layer. Figure S2. XPS survey spectra of the pristine β-PbO_2_, Mn-PbO_2_ and LaMn-PbO_2_ electrodes. Figure S3. SEM images of the prepared electrodes: β-PbO_2_, Mn-PbO_2_ and LaMn-PbO_2_.
