# Supplementary material for: Defect-Engineered La-Mn Co-Doped β-PbO2 Anodes for Energy-Efficient Zinc Electrowinning
Source: Materials (Basel). 2026 Mar 30;19(7):1370. doi: 10.3390/ma19071370 (PMC13074564; doi:10.3390/ma19071370)
Supplement: Supplementary file 1 [file materials-19-01370-s001.zip › materials-4212054-supplementary.pdf]

Defect-Engineered La-Mn Co-doped β-PbO_2 Anodes for Energy-Efficient Zinc Electrowinning

Yi Luo, Nan Li, Lingjing Yang , Jinlong Wei , Yuantao Yang ,Wentao Wang ,YangZhao ,Ruidong Xu , Xuanbing Wang

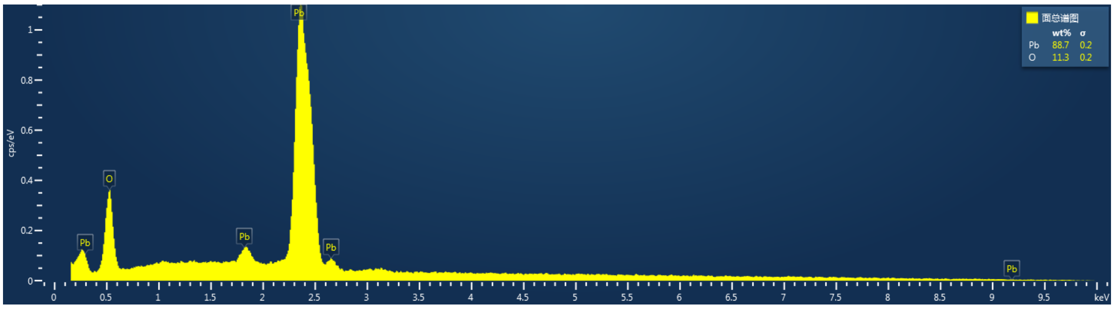

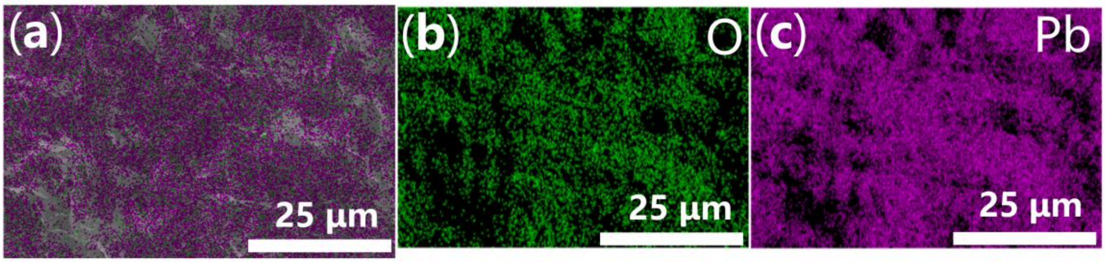

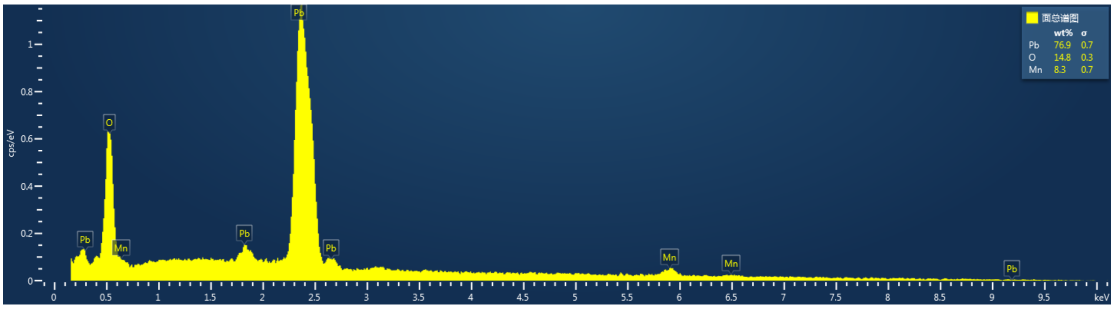

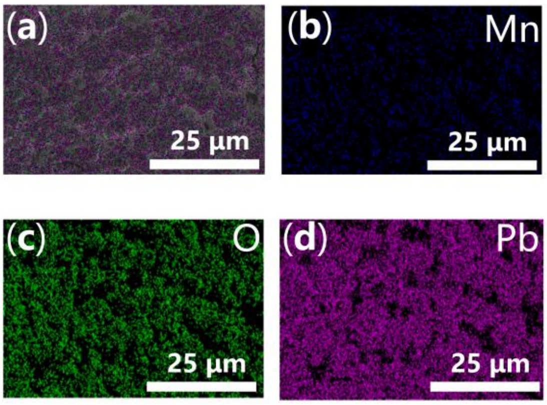

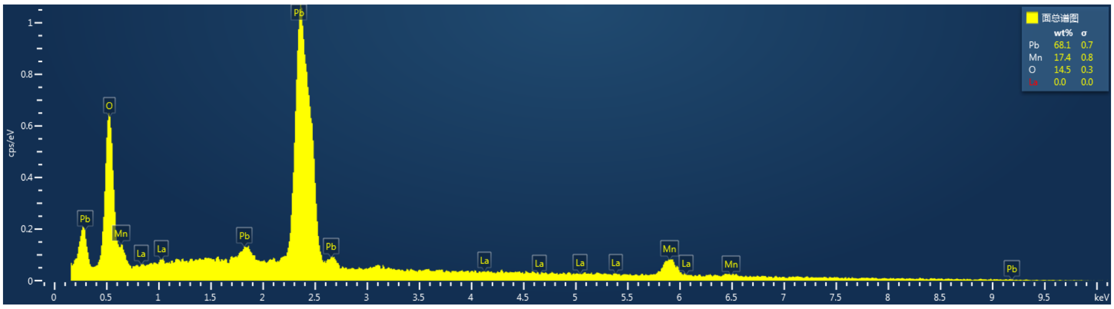

Figure S1. EDS elemental mapping (Pb, O, Mn, and La) of (a) β-PbO_2, (b) Mn-PbO_2, and (c) LaMn-PbO_2 electrodes.

Table S1. Comparison of interfacial capacitance parameters (C_{eff}) for the prepared electrodes in acidic zinc electrowinning electrolyte.

**C_{dl}(mF)**	**β-PbO_2**	**Mn-PbO_2**	**LaMn- PbO_2**
**1.0 V**	0.0088	1.30	21.32
**1.1 V**	0.063	2.42	11.37
**1.2 V**	0.0337	5.30	10.12
**1.3 V**	0.0627	6.91	1.68

Table S2. Fitting parameters of the equivalent circuit ($R_s(QR_{ct})$) for different anodes at various anodic potentials in the zinc electrowinning electrolyte.

**R_{ct}(Ω)**	**β-PbO_2**	**Mn-PbO_2**	**LaMn- PbO_2**
**1.0 V**	147.6	264.2	34.72
**1.1 V**	141.3	54.3	44.29
**1.2 V**	149.5	11.5	15.72
**1.3 V**	80.3	4.1	13.93

Table S3. Characteristic frequency values (f_{max}) determined from Bode phase plots for different anodes at various anodic potentials in the zinc electrowinning electrolyte.

**F_{max}(Hz)**	**β-PbO_2**	**Mn-PbO_2**	**LaMn- PbO_2**
**1.0 V**	122	0.464	0.215
**1.1 V**	17.8	1.21	0.316
**1.2 V**	31.6	2.61	1
**1.3 V**	31.6	5.62	6.81

Table S4. corresponding relaxation time constants (τ) for the prepared anodes at different anodic potentials. in the zinc electrowinning electrolyte.

τ(s)[a]	β-PbO_2	Mn-PbO_2	LaMn- PbO_2
1.0 V	$1.30\ 10^{-3}$	0.343	0.740
1.1 V	$8.94\ 10^{-3}$	0.132	0.504
1.2 V	$5.04\ 10^{-3}$	0.061	0.159
1.3 V	$5.04\ 10^{-3}$	0.028	0.023

Table S5. Proposed electrochemical deposition mechanism of PbO_2 active layer.

Step	Chemical Reaction Equation	**No.**
Water dissociation	$H_2O \rightarrow {*OH} + H^2 + e^-$	(1)
Intermediate formation	${*OH} + Pb^{2+} \rightarrow Pb(OH)^{2+}$	(2)
Final oxidation	$Pb(OH)^{2+} + H_2O \rightarrow PbO_2 + 3H^+ + e^-$	(3)

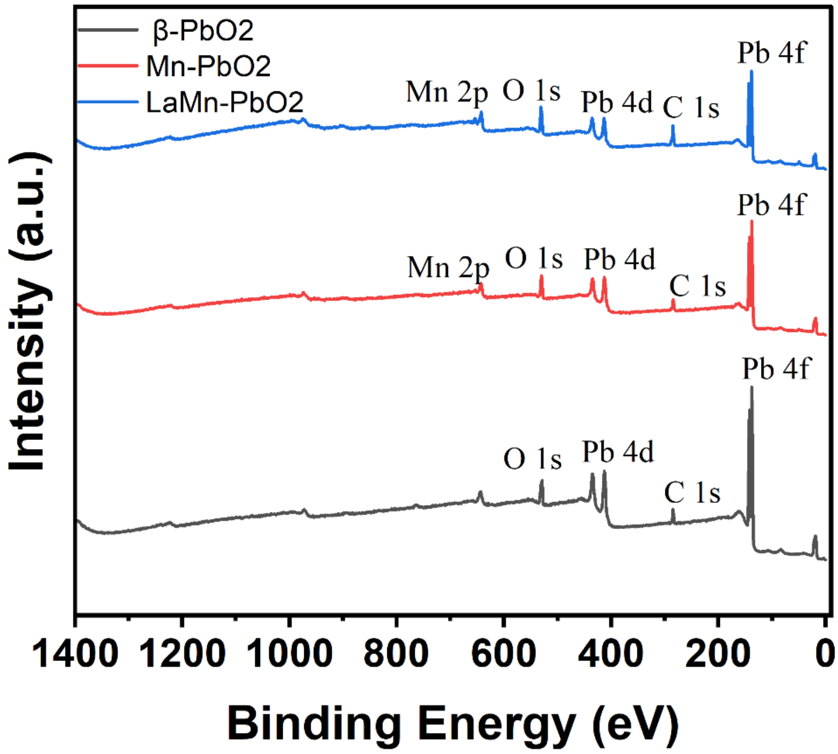

Figure S2. XPS survey spectra of the pristineβ-PbO_2, Mn-PbO_2 and LaMn-PbO_2 electrodes.

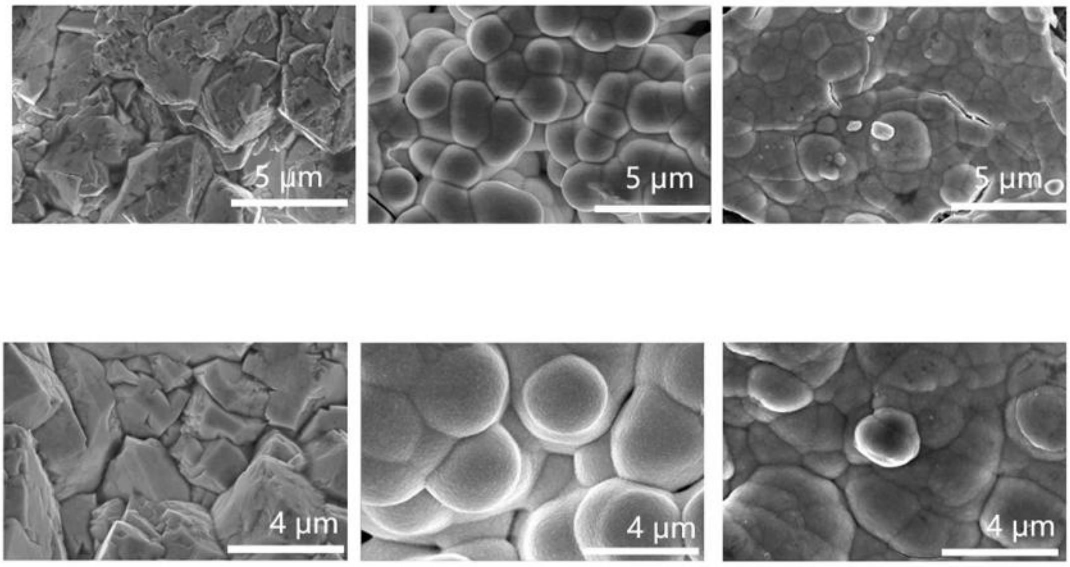

Figure S3. SEM images of the prepared electrodes: β-PbO_2 , Mn-PbO_2 and LaMn-PbO_2
